# Deaths of despair before and during the COVID-19 pandemic: a comparative analysis of 27 European countries and the United States

**DOI:** 10.3389/fpubh.2026.1818398

**Published:** 2026-05-21

**Authors:** Ronny Westerman, Michael Mühlichen

**Affiliations:** Federal Institute for Population Research, Wiesbaden, Germany

**Keywords:** alcohol-related deaths, COVID-19, deaths of despair, drug-related deaths, European countries, suicide, United States

## Abstract

**Background:**

The phenomenon of “deaths of despair” has been frequently described in the context of the United States but has rarely been analyzed across multiple countries. The onset of the coronavirus disease 2019 (COVID-19) pandemic, accompanied by the imposition of substantial restrictions by public authorities, may have worsened mental health at the individual level, thereby potentially increasing the number of despair-related deaths.

**Objective:**

This study investigates trends and differences in despair-related deaths across 27 European countries and the United States before and during the COVID-19 pandemic.

**Methods:**

Using official cause-of-death statistics, we estimated standardized death rates (SDRs) by country and sex from 2011–2021 for despair-related deaths and five sub-categories based on a harmonized classification. To quantify the relative change during the two pandemic years (2020-2021) compared to the five preceding years (2015–2019), we calculated standardized mortality ratios (SMRs).

**Results:**

In most European countries, the number of despair-related deaths remained stable or declined over the past decade. However, this downward trend did not continue during the pandemic. Increases were observed in Eastern European countries and Ireland, but remained well below those in the United States.

**Contribution:**

This study is the first to explore recent trends in deaths of despair across wide parts of Europe in comparison with the United States based on a harmonized classification. The underlying causes of these deaths, which are considered preventable, are multifaceted and necessitate the implementation of targeted public health strategies.

## 1 Introduction

The rise in suicide and drug- and alcohol-related deaths among young and middle-aged males in several high-income countries since the late 1990s has attracted substantial attention in public health research (1–4). These causes of death are commonly subsumed under *deaths of despair* and linked to adverse social and economic changes, including deteriorating labor market conditions (e.g., structural change, mass unemployment, recessions) and the erosion of stabilizing social institutions such as religion, family, marriage, and trade unions. These processes may contribute to a loss of meaning in life among vulnerable individuals, which is often accompanied by unhealthy behaviors and elevated risks of premature mortality (5–7).

Rising deaths of despair were long considered largely confined to the United States. However, more recent evidence indicates that similar trends were observed in other high-income countries well before the COVID-19 pandemic (1, 3, 8–11).

The COVID-19 pandemic, as a natural experiment, constituted a major societal shock, with far-reaching consequences for mental health and health-related behaviors. Stringent containment measures—including lockdowns, school closures, job losses, and social isolation—were associated with increased risks of mental health problems (12) and higher levels of hazardous alcohol consumption and illicit drug use (13, 14).

Although some studies suggest that illicit drug use declined during the pandemic, this does not necessarily imply a reduction in harmful use or fatal overdoses (15, 16). To date, empirical evidence on pandemic-related changes in deaths of despair has focused mainly on the United States and the United Kingdom.

Recent studies reported increases in alcohol-related mortality in both countries and a pronounced rise in drug-related deaths in the United States, while suicide mortality increased only slightly (17). Evidence from other European countries remains scarce. For example, a study from Spain documented long-term trends in deaths of despair but was limited to the pre-pandemic period (3). Overall, existing literature largely focuses on the early phase of the pandemic and selected countries.

This raises the question of how deaths of despair evolved in other European countries, particularly those that experienced more severe impacts of the COVID-19 pandemic and implemented comparatively stringent containment measures. Early-pandemic population surveys reported deteriorating mental health, with increased depression, anxiety, and suicidal ideation, especially among younger adults (18–22).

Following lockdowns and mobility restrictions, individuals with substance use disorders were affected by reduced access to established drug supplies and substitution treatment (23).

In addition, economic strain during the pandemic may have exacerbated vulnerabilities, while the closure of bars and restaurants likely shifted alcohol consumption toward home drinking as a coping strategy (23).

Cross-national differences in lockdown intensity and social distancing policies were associated with reductions in life satisfaction across Europe, depending on policy stringency (24). Although it remains unclear whether stricter containment measures directly increased suicide risk (12) deteriorations in well-being and mental health may have contributed indirectly to increases in harmful alcohol and drug use and, ultimately, to higher mortality from these causes (25, 26).

Against this background, it remains unclear how deaths of despair evolved across European countries before and during the COVID-19 pandemic and how these trends compare with those observed in the United States. Cross-country comparisons are further complicated by conceptual differences in the interpretation of deaths of despair, particularly between the United States and Eastern Europe (27). Using a harmonized classification of deaths of despair based on official cause-specific mortality data, this study therefore examines trends in alcohol-, drug-, and suicide-related mortality in 27 European countries and the United States before and during the COVID-19 pandemic.

## 2 Data and methods

### 2.1 Data

We used cause-specific death counts for all selected countries according to the 10th revision of the International Classification of Diseases (ICD-10) for 2011–2021, by sex and 5-year age groups (0–14, 15–24, 25–29, …, 90–94, 95+ years). Data for European countries were obtained from Eurostat, and data for the United States from the Centers for Disease Control and Prevention (CDC) via the CDC WONDER database. Corresponding population estimates were retrieved from Eurostat (for the European countries) and the Human Mortality Database (for the United States).

We selected 27 European countries with available cause-specific death counts for every year of the study period and populations of at least one million: Austria, Belgium, Bulgaria, Croatia, Czechia, Denmark, Estonia, Finland, France, Germany, Greece, Hungary, Ireland, Italy, Latvia, Lithuania, Netherlands, Norway, Poland, Portugal, Romania, Serbia, Slovakia, Slovenia, Spain, Sweden, and Switzerland. Data were derived from mandatory medical death certificates, following World Health Organization (WHO) coding rules.

The United Kingdom was excluded due to unavailability of cause-of-death data in Eurostat after 2019. Hence, comparability with EU member states cannot be maintained.

### 2.2 Concept of “deaths of despair”

There are notable terminological differences in the use of the term *deaths of despair*. The most commonly cited definition (1) includes suicide (X60–84, Y87.0), poisoning (X40–45; Y10–15, Y45, Y47, Y49), and alcoholic liver disease and cirrhosis (K70, K73-74). Poisoning encompasses both accidental and intentional deaths due to alcohol or drug overdose, including prescriptions and illicit drugs, which may lead to over- or underestimation of specific causes.

Alternatively, Angus et al. (17) define deaths of despair more specifically as suicide (U03, X60–84, Y87), alcohol-related (K70, K73–74, F10, X45, Y15), and drug-related (F11–16, F18–19, X40–44, X85, Y10–14) deaths, emphasizing alcohol- and drug-related mortality. This definition is suitable for United States–United Kingdom comparisons.

To ensure compatibility with Eurostat cause-of-death categories, we applied an adjusted classification for all countries studied, comprising five subgroups: alcohol-related mental and behavioral disorders (F10), chronic liver disease (K70, K73-K74), drug-related mental and behavioral disorders (F11–F16, F18-F19), unintentional poisoning and exposure to harmful substances (X40–X49), and suicide (X60–X84; Y10–Y34, Y87.0, Y87.2). Events of undetermined intent were included in the suicide category due to considerable cross-country coding differences (28). Further differentiation of deaths of despair, particularly alcohol-related deaths as performed by Allik et al. (5), is not possible with Eurostat data.

### 2.3 Statistical analysis

We estimated standardized death rates (SDRs) for deaths of despair and each of the five sub-cause groups for males and females and for both sexes combined. SDRs were calculated using the 2013 European Standard Population. To quantify the relative change in the observed countries between the COVID-19 pandemic years (2020-2021) and the five pre-pandemic years (2015–2019), we calculated standardized mortality ratios (SMRs). Estimates were extracted separately for males and females, as well as for specific age groups. All estimates of SDRs and SMRs and their 95% confidence intervals were derived using R Studio 4.1.2 software (29).

## 3 Results

**Figure 1:**
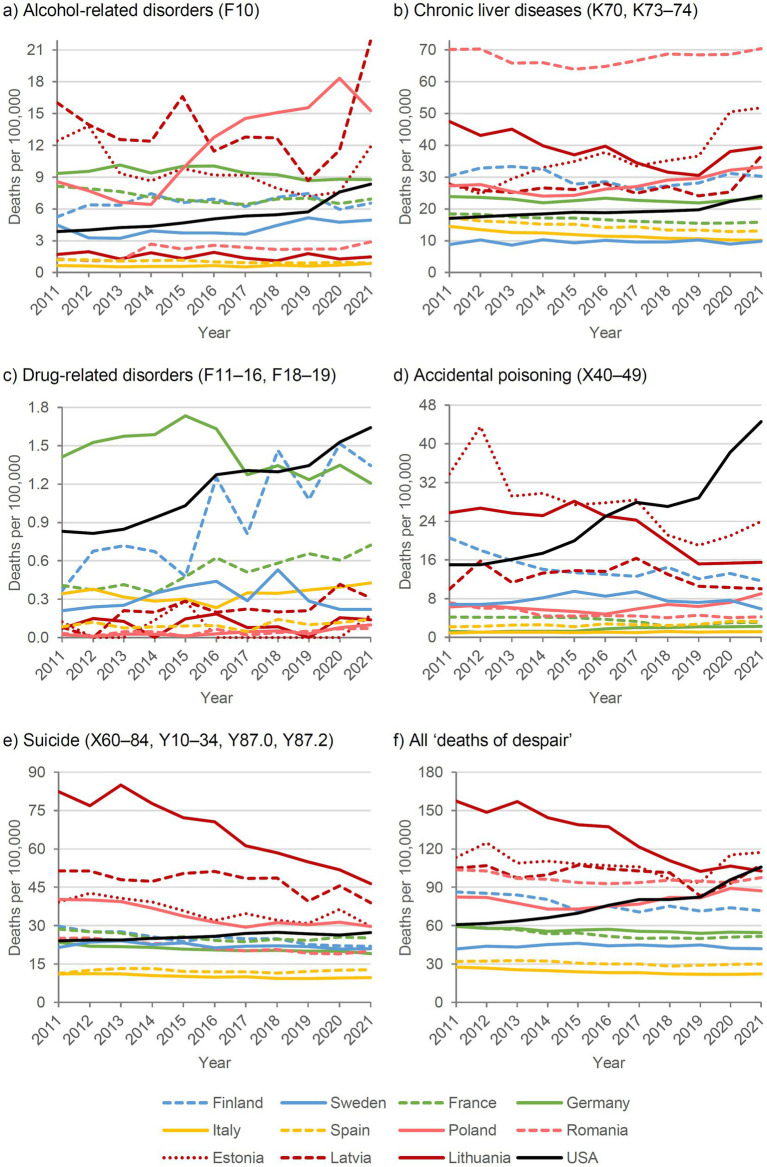
Deaths of despair by cause and country, 2011–2021, standardized death rate, males.

**Figure 2:**
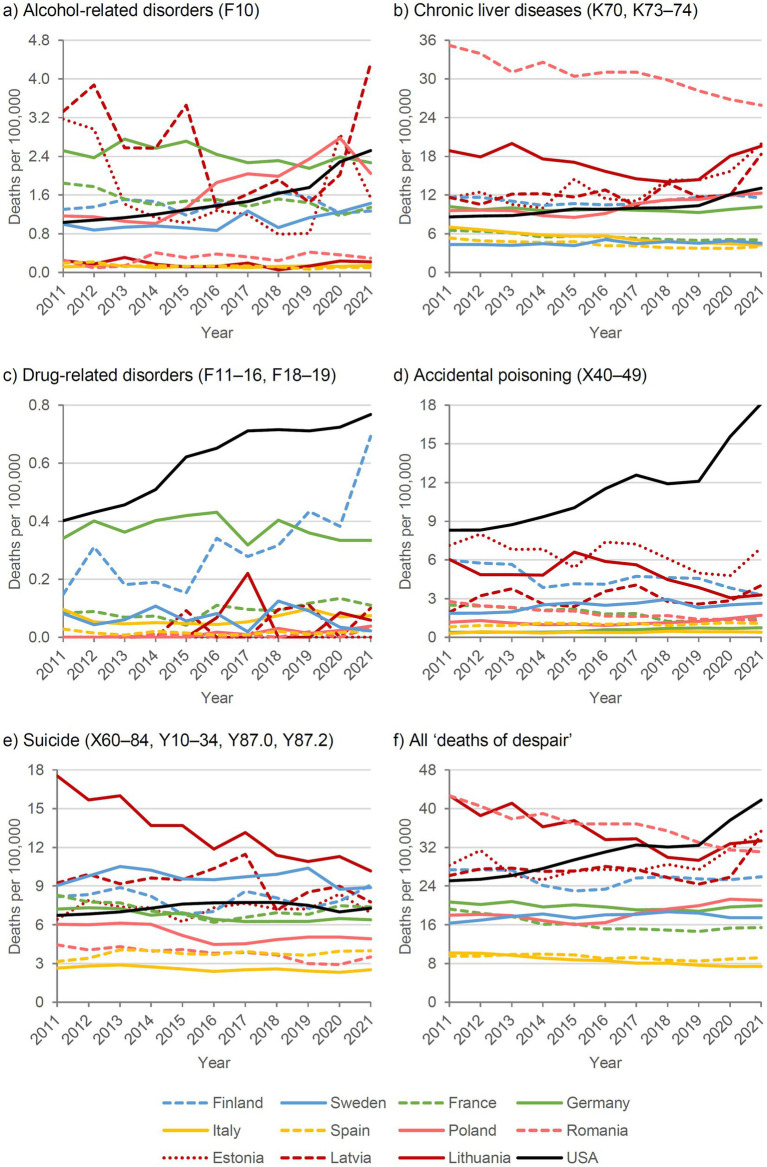
Deaths of despair by cause and country, 2011–2021, standardized death rate, females.

Figures 1 and 2 show the SDRs for deaths of despair in the selected countries for males and females, respectively, with Northern European countries in blue, Western European countries in green, Southern European countries in yellow, Eastern European countries in red (Baltic states in dark red), and the United States in black.

**Figure 3:**
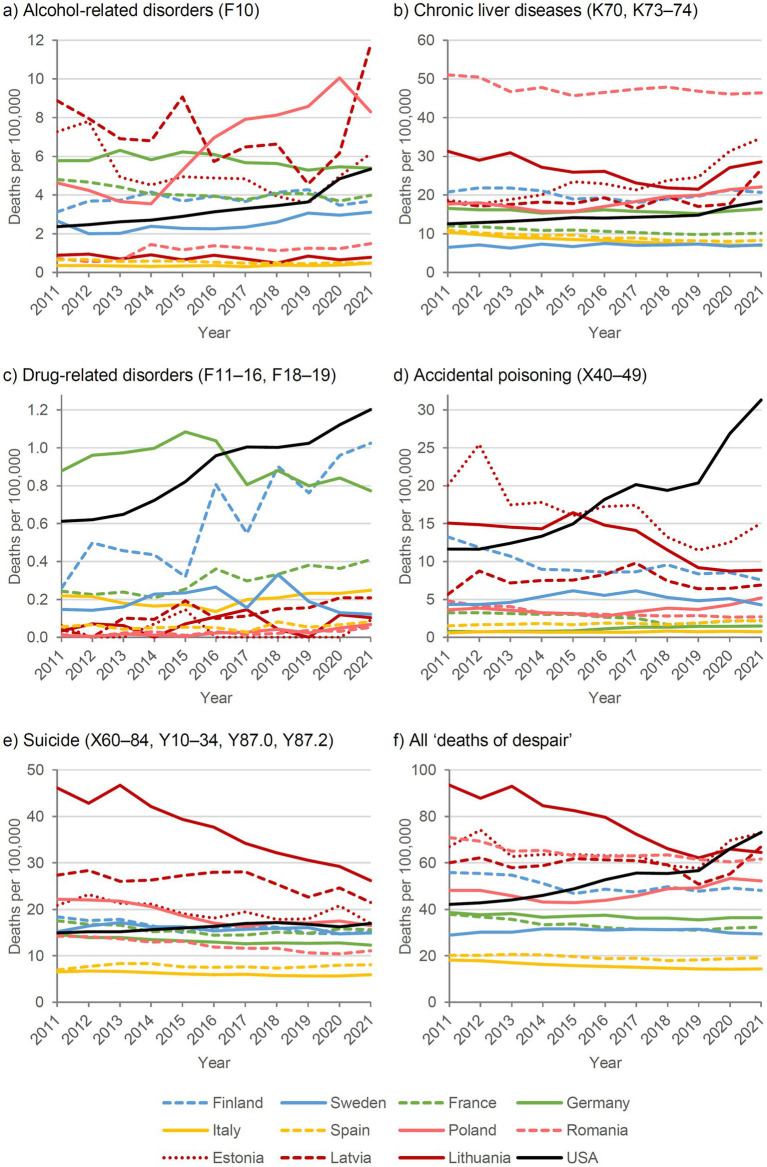
Deaths of despair by cause and country, 2011–2021, standardized death rate, both sexes.

SDRs for both sexes combined are shown in Figure 3. For clarity, the figures show only selected countries. Complete estimates of SDRs and SMRs for all 28 countries are provided in the Supplementary material, together with sensitivity analyses for suicides excluding events of undetermined intent, SMRs using 2019 as the baseline year, and proportional mortality ratios.

In most European countries, a stagnant or decreasing trend in deaths of despair was observed over the past decade, except in Poland and the Baltic states of Estonia and Latvia. In these countries, the decreasing trend did not continue during the COVID-19 pandemic years 2020-2021. In Eastern Europe, particularly Lithuania, deaths of despair declined between 2011 and 2019, whereas in the United States they increased markedly among both males and females until 2016, followed by a period of stagnation between 2017 and 2019.

With the onset of the COVID-19 pandemic in 2020, SDRs began increasing again in the United States. Some European countries, such as Lithuania and Poland, also experienced slight increases in 2020 and 2021, although to a substantially lesser extent than in the United States. Countries in Southern Europe, including Italy and Spain, maintained very low levels of despair-related mortality even during the COVID-19 pandemic, despite implementing the most restrictive policies with prolonged lockdowns compared with other European countries. In Germany and France, which had moderate policy restrictions, and Sweden, which had the least restrictive policies, deaths of despair remained mostly stable, with only marginal increases (Germany and France) or decreases (Sweden) in 2020 and 2021.

Regarding the five sub-causes, suicides increased in Estonia, Latvia, and Ireland in 2020, and in Slovenia in 2021, but did not deviate notably from the long-term trend in the other observed countries. Alcohol-related mortality, both due to chronic liver diseases and mental/behavioral disorders, showed the largest increase, particularly in Eastern European countries. Trends in drug-related mortality remained relatively stable in most countries. However, notable increases during the two pandemic years were observed in Estonia and Poland (due to accidental poisonings) and in Slovenia and Finland (due to drug-related mental/behavioral disorders), and in particular in the United States due to both sub-causes.

Comparing males and females, a persistent gap in the risk of deaths of despair was observed, with males consistently at higher risk. This male disadvantage was apparent across all subcategories, including suicide, drug-related, and alcohol-related deaths, and was most pronounced in Eastern European countries.

**Figure 4:**
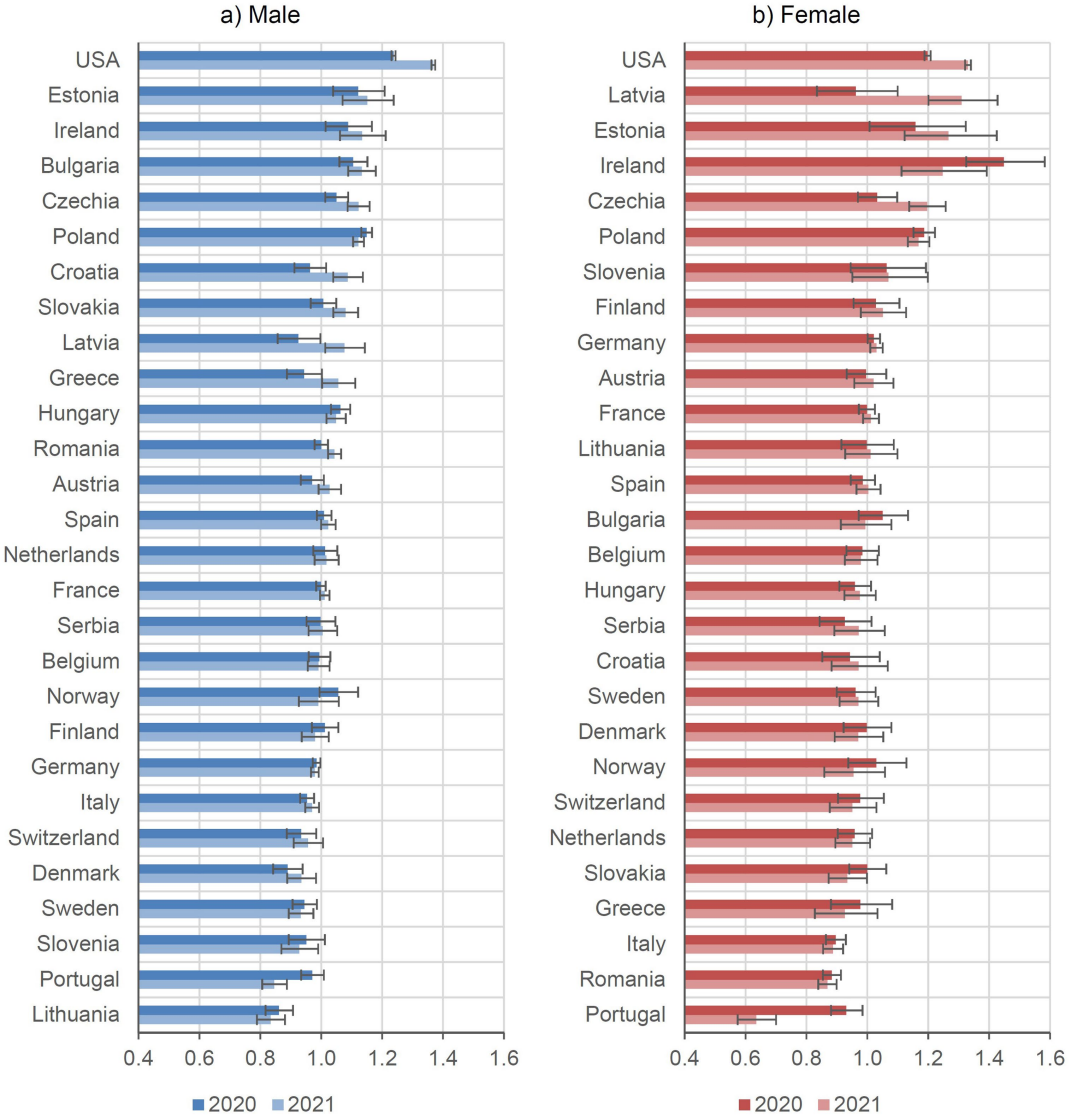
Deaths of despair (all sub-causes combined) for 2020 and 2021, standardized mortality ratio (with years 2015–2019 as baseline) with 95% confidence intervals by sex and country.

As illustrated in Figure 4, SMRs for deaths of despair showed a markedly heterogeneous pattern across European countries and the United States for both sexes. While some countries demonstrated increasing trends during the COVID-19 pandemic, others showed stable or declining trends.

Cross-country comparison revealed a pronounced increase in deaths of despair in the United States for 2020 and 2021 relative to the pre-pandemic period (2015–2019). In 2020, SMRs were 1.24 for males and 1.20 for females, corresponding to increases of 24 and 20%, respectively. In 2021, SMRs rose to 1.37 for males and 1.33 for females (increases of 37 and 33%).

Some Eastern European countries and Ireland also showed increases, albeit smaller than in the United States. Estonia had the most pronounced increase among Eastern European countries, with SMRs of 1.12 for males and 1.16 for females in 2020, rising to 1.15 and 1.27 in 2021. In Ireland, male SMRs increased moderately from 1.09 in 2020 to 1.13 in 2021, while female SMRs declined from 1.45 to 1.25 over the same period.

Sweden was among the few countries with declining SMRs during the pandemic: for males, SMRs were 0.95 in 2020 and 0.93 in 2021 (decreases of 5 and 7%), and for females, 0.96 and 0.97 (decreases of 4 and 3%). Portugal also had exceptionally low SMRs during the pandemic, with male SMRs of 0.97 and 0.84 (reductions of 3 and 16%) and female SMRs of 0.93 and 0.64 (reductions of 7 and 36%).

## 4 Discussion

The COVID-19 pandemic and stringent government control measures substantially affected mental health and mortality patterns. From the early stages of the pandemic, the negative effects of social isolation and other restrictive measures on mental health were widely discussed (12, 30, 31). This study examined how trends in deaths of despair (related to alcohol, drugs, and suicide) before and during the COVID-19 pandemic differed between European countries and the United States.

Across countries, the United States clearly stood out as the most pronounced outlier, showing substantially larger increases in deaths of despair during the pandemic than any European country. A notable exception was observed for Irish females in 2020. Apart from the United States and Ireland, increases during the pandemic years were also observed in several Eastern European countries, most pronouncedly in Estonia, largely driven by alcohol-related mortality, whereas most Western and Southern European countries exhibited stable or only marginal changes. Some countries, such as Sweden, Italy and Portugal, even showed declines in both sexes, reflecting disparate impacts of the pandemic across countries.

Our results indicated only a modest increase in suicide deaths, with variation across countries. The sharp rise in alcohol-related deaths since 2020 suggests increased alcohol consumption during the pandemic in several countries (32, 33). Trends in drug-related mortality remained relatively stable in most countries, with only minor changes from pre-pandemic levels. Severe contact restrictions and lockdowns may have reduced the supply of illicit drugs (34, 35) potentially leading to increased alcohol use as a substitute.

Strengths of this study include the use of official cause-of-death data from Eurostat and CDC WONDER to classify deaths of despair consistently across countries and over time. Applying this classification facilitated cross-country comparisons of despair-related mortality trends between Europe and the United States before and during the COVID-19 pandemic.

Several limitations should be noted. First, despite the harmonized classification, national coding differences remain, especially for drug-related deaths. While not all classified deaths are necessarily related to despair, the concept has been criticized for overlooking additional causes of death that may be indirectly influenced by psychosocial distress, such as obesity, cancer, diabetes, and cardiovascular disease (36, 37).

Second, the present analyses are descriptive and do not permit causal attribution of changes observed during 2020-2021 to the COVID-19 pandemic or to specific policy responses. Country-level contextual factors such as economic conditions, income inequality, healthcare capacity, and the stringency of containment measures were not incorporated and may partly explain cross-national differences in levels and trends. In addition, social gradients could not be considered. Prior research has shown higher despair-related mortality among individuals with lower education, insecure employment, and disadvantaged living conditions (38–41).

## 5 Conclusion

This study is the first to provide a harmonized comparative assessment of deaths of despair in the United States and a broad set of European countries before and during the COVID-19 pandemic. Overall, levels and trends are most favorable in Western Europe, particularly southern countries (Italy and Spain), while high levels and increases during the COVID-19 pandemic in Eastern Europe are largely driven by alcohol-related deaths. In the United States, by contrast, drug-related deaths, particularly accidental poisonings, contributed to increases before (up to 2017) and during the pandemic, exceeding despair-related mortality in most Eastern European countries.

Despair-related deaths are generally considered avoidable through primary prevention (42, 43). Their underlying causes—as reflected in the heterogeneous patterns across the five sub-categories—are multifaceted, vary by national and regional context, and require targeted public health strategies (44). Future research should extend analyses to the regional level and complement descriptive monitoring with causal inference approaches using longitudinal and contextual area-level data to better understand the drivers of cross-national and subnational differences and changes during public health crises.

## Data Availability

The datasets supporting the conclusions of this article are publicly available. European cause-of-death data can be obtained from the Eurostat database of the European Union: https://doi.org/10.2908/HLTH_CD_ARO. Corresponding population counts are also available at Eurostat: https://doi.org/10.2908/DEMO_PJAN. American cause-of-death data are accessible at the CDC WONDER database produced by National Center for Health Statistics (NCHS) at the Centers for Disease Control and Prevention (CDC): https://wonder.cdc.gov/deaths-by-underlyingcause.html. Corresponding population counts are available at the Human Mortality Database: https://www.mortality.org.

## References

[1] Case A Deaton A. Rising morbidity and mortality in midlife among white non-Hispanic Americans in the 21st century. Proc Natl Acad Sci USA. (2015) 112:15078–83. doi: 10.1073/pnas.1518393112, 26575631 PMC4679063

[2] Erwin PC. Despair in the American heartland? A focus on rural health. Am J Public Health. (2017) 107:1533–4. doi: 10.2105/AJPH.2017.304029, 28902542 PMC5607702

[3] Piñeiro B Spijker JJA Trias-Llimós S Blanes Llorens A Permanyer I. Trends in cause-specific mortality: deaths of despair in Spain, 1980–2019. J Public Health. (2023) 45:854–62. doi: 10.1093/fdad1133 PMC10687877 37491646

[4] Stein EM Gennuso KP Ugboaja DC Remington PL. The epidemic of despair among white Americans: trends in the leading causes of premature death, 1999–2015. Am J Public Health. (2017) 107:1541–7. doi: 10.2105/AJPH.2017.303941, 28817333 PMC5607670

[5] Allik M Brown D Dundas R Leyland AH. Deaths of despair: cause-specific mortality and socioeconomic inequalities in cause-specific mortality among young men in Scotland. Int J Equity Health. (2020) 19:215. doi: 10.1186/s12939-020-01329-7, 33276793 PMC7716282

[6] Case A Deaton A. The epidemic of despair: will America’s mortality crisis spread over the world? Foreign Aff. (2020) 99:92–102.

[7] Case A Deaton A. The great divide: education, despair, and death. Annu Rev Econ. (2022) 14:1–21. doi: 10.1146/annurev-economics-051520-015607, 35990244 PMC9389919

[8] Sterling P Platt ML. Why deaths of despair are increasing in the US and not other industrial nations—insights from neuroscience and anthropology. JAMA Psychiatry. (2022) 79:368–74. doi: 10.1001/jamapsychiatry.2021.4209, 35107578

[9] Steelesmith DL Lindstrom MR Le HTK Root ED Campo JV Fontanella CA. Spatiotemporal patterns of deaths of despair across the U.S., 2000–2019. Am J Prev Med. (2023) 65:192–200. doi: 10.1016/j.amepre.2023.02.020, 36964010

[10] Becchetti L Conzo G. Avoiding a ‘despair death crisis’ in Europe: the drivers of human (un)sustainability. Int Rev Econ. (2021) 68:485–526. doi: 10.1007/s12232-021-00379-9

[11] Dowd JB Angus C Zajacova A Tilstra AM. Comparing trends in mid-life ‘deaths of despair’ in the USA, Canada and UK, 2001–2019: is the USA an anomaly? BMJ Open. (2023) 13:e069905. doi: 10.1136/bmjopen-2022-069905, 37591647 PMC10441077

[12] Aknin LB Andretti B Goldszmidt R Helliwell JF Petherick A De Neve JE. Policy stringency and mental health during the COVID-19 pandemic: a longitudinal analysis of data from 15 countries. Lancet Public Health. (2022) 7:e417–26. doi: 10.1016/S2468-2667(22)00060-3, 35461592 PMC9023007

[13] Dogan-Sander E Kohls E Baldofski S Rummel-Kluge C. More depressive symptoms, alcohol and drug consumption: increase in mental health symptoms among university students after one year of the COVID-19 pandemic. Front Psych. (2021) 12:790974. doi: 10.3389/fpsyt.2021.790974, 34975589 PMC8716753

[14] Weerakoon SM Jetelina KK Knell G Messiah SE. COVID-19 related employment change is associated with increased alcohol consumption during the pandemic. Am J Drug Alcohol Abuse. (2021) 47:730–6. doi: 10.1080/00952990.2021.1912063, 34043919

[15] Been F Emke E Matias J Baz-Lomba JA Boogaerts T Castiglioni S . Changes in drug use in European cities during early COVID-19 lockdowns - a snapshot from wastewater analysis. Environ Int. (2021) 153:106540. doi: 10.1016/j.envint.2021.106540, 33838618 PMC7997602

[16] Vo AT Patton T Peacock A Larney S Borquez A. Illicit substance use and the COVID-19 pandemic in the United States: a scoping review and characterization of research evidence in unprecedented times. Int J Environ Res Public Health. (2022) 19:8883. doi: 10.3390/ijerph19148883, 35886734 PMC9317093

[17] Angus C Buckley C Tilstra AM Dowd JB. Increases in ‘deaths of despair’ during the COVID-19 pandemic in the United States and the United Kingdom. Public Health. (2023) 218:92–6. doi: 10.1016/j.puhe.2023.02.019, 36996743 PMC9968617

[18] Berger K Riedel-Heller S Pabst A Rietschel M Richter D NAKO-Konsortium. Einsamkeit während der ersten Welle der SARS-CoV-2-Pandemie: Ergebnisse der NAKO-Gesundheitsstudie [loneliness during the first wave of the SARS-CoV-2 pandemic-results of the German National Cohort (NAKO)]. Bundesgesundheitsbl. (2021) 64:1157–64. doi: 10.1007/s00103-021-03393-y, 34327541 PMC8320420

[19] Bu F Steptoe A Fancourt D. Who is lonely in lockdown? Cross-cohort analyses of predictors of loneliness before and during the COVID-19 pandemic. Public Health. (2020) 186:31–4. doi: 10.1016/j.puhe.2020.06.036, 32768621 PMC7405905

[20] Dragano N Reuter M Peters A Engels M Schmidt B Greiser KH . Increase in mental disorders during the COVID-19 pandemic—the role of occupational and financial strains. An analysis of the German National Cohort (NAKO) study. Dtsch Arztebl Int. (2022) 119:179–87. doi: 10.3238/arztebl.m2022.0133, 35197188 PMC9229580

[21] Groarke JM Berry E Graham-Wisener L McKenna-Plumley PE McGlinchey E Armour C. Loneliness in the UK during the COVID-19 pandemic: cross-sectional results from the COVID-19 psychological wellbeing study. PLoS One. (2022) 15:e0239698. doi: 10.1371/journal.pone.0239698, 32970764 PMC7513993

[22] Hoffart A Johnson SU Ebrahimi OV. Loneliness and social distancing during the COVID-19 pandemic: risk factors and associations with psychopathology. Front Psych. (2020) 11:589127. doi: 10.3389/fpsyt.2020.589127, 33329136 PMC7714714

[23] Schecke H Bohn A Scherbaum N Mette C. Alcohol use during COVID-19 pandemic on the long run: findings from a longitudinal study in Germany. BMC Psychol. (2022) 10:266. doi: 10.1186/s40359-022-00965-8, 36376933 PMC9661459

[24] Clark AE Lepinteur A. Pandemic policy and life satisfaction in Europe. Rev Income Wealth. (2022) 68:393–408. doi: 10.1111/roiw.12554, 34908597 PMC8661917

[25] Jacob L Smith L Armstrong NC Yakkundi A Barnett Y Butler L . Alcohol use and mental health during COVID-19 lockdown: a cross-sectional study in a sample of UK adults. Drug Alcohol Depend. (2021) 219:108488. doi: 10.1016/j.drugalcdep.2020.108488, 33383352 PMC7768217

[26] Roberts A Rogers J Mason R Siriwardena AN Hogue T Whitley GA . Alcohol and other substance use during the COVID-19 pandemic: a systematic review. Drug Alcohol Depend. (2021) 229:109150. doi: 10.1016/j.drugalcdep.2021.109150, 34749198 PMC8559994

[27] King L Scheiring G Nosrati E. Deaths of despair in comparative perspective. Annu Rev Sociol. (2022) 48:299–317. doi: 10.1146/annurev-soc-030320-031757

[28] Danilova I Rau R Barbieri M Grigoriev P Jdanov DA Meslé F . Subnational consistency in cause-of-death data: the cases of Russia, Germany, the United States, and France. Population. (2021) 76:645–74. doi: 10.3917/popu.2104.0693

[29] R Core Team. R: A Language and Environment for Statistical Computing. Vienna: R Foundation for Statistical Computing (2024).

[30] Salanti G Peter N Tonia T Holloway A White IR Darwish L . The impact of the COVID-19 pandemic and associated control measures on the mental health of the general population: a systematic review and dose-response meta-analysis. Ann Intern Med. (2022) 175:1560–71. doi: 10.7326/M22-1507, 36252247 PMC9579966

[31] Fernández D Giné-Vázquez I Morena M Koyanagi AI Janko MM Haro JM . Government interventions and control policies to contain the first COVID-19 outbreak: an analysis of evidence. Scand J Public Health. (2023) 51:682–91. doi: 10.1177/14034948231156969, 36883722 PMC9996153

[32] Calina D Hartung T Mardare I Mitroi M Poulas K Tsatsakis A . COVID-19 pandemic and alcohol consumption: impacts and interconnections. Toxicol Rep. (2021) 8:529–35. doi: 10.1016/j.toxrep.2021.03.005, 33723508 PMC7944101

[33] Kilian C O’Donnell A Potapova N López-Pelayo H Schulte B Miquel L . Changes in alcohol use during the COVID-19 pandemic in Europe: a meta-analysis of observational studies. Drug Alcohol Rev. (2022) 41:918–31. doi: 10.1111/dar.13446 35187739 PMC9111882

[34] Scherbaum N Bonnet U Hafermann H Schifano F Bender S Grigoleit T . Availability of illegal drugs during the COVID-19 pandemic in western Germany. Front Psych. (2021) 12:648273. doi: 10.3389/fpsyt.2021.648273, 33967857 PMC8102785

[35] Gardner EA McGrath SA Dowling D Bai D. The opioid crisis: prevalence and markets of opioids. Forensic Sci Rev. (2022) 34:43–70. 35105535

[36] Liao B Xu D Tan Y Chen X Cai S. Association of mental distress with chronic diseases in 1.9 million individuals: a population-based cross-sectional study. J Psychosom Res. (2022) 162:111040. doi: 10.1016/j.jpsychores.2022.111040, 36137487

[37] Song J Kang S Ryff CD. Unpacking psychological vulnerabilities in deaths of despair. Int J Environ Res Public Health. (2023) 20:6480. doi: 10.3390/ijerph20156480, 37569020 PMC10418686

[38] Beseran E Pericàs JM Cash-Gibson L Ventura-Cots M Porter KMP Benach J. Deaths of despair: a scoping review on the social determinants of drug overdose, alcohol-related liver disease and suicide. Int J Environ Res Public Health. (2022) 19:12395. doi: 10.3390/ijerph191912395, 36231697 PMC9566538

[39] Kuo CT Kawachi I. County-level income inequality, social mobility, and deaths of despair in the US, 2000–2019. JAMA Netw Open. (2023) 6:e2323030. doi: 10.1001/jamanetworkopen.2023.23030, 37436752 PMC10339154

[40] Camacho C Webb RT Bower P Munford L. Risk factors for deaths of despair in England: an ecological study of local authority mortality data. Soc Sci Med. (2024) 342:116560. doi: 10.1016/j.socscimed.2024.116560, 38215641

[41] Lübker C Murtin F. Educational inequalities in deaths of despair in 14 OECD countries: a cross-sectional observational study. J Epidemiol Community Health. (2025) 79:75–81. doi: 10.1136/jech-2024-222089, 39019490 PMC11874332

[42] Mühlichen M Lerch M Sauerberg M Grigoriev P. Different health systems - different mortality outcomes? Regional disparities in avoidable mortality across German-speaking Europe, 1992–2019. Soc Sci Med. (2023) 329:115976. doi: 10.1016/j.socscimed.2023 37356189 PMC10357323

[43] Stroisch S Mühlichen M Grigoriev P Vogt T. Spatial differences in avoidable mortality across 581 European districts, 2002–2019. Eur J Popul. (2026) 42:5. doi: 10.1007/s10680-025-09761-7, 41364281 PMC12791106

[44] Lanfiuti Baldi G Nigri A Trias-Llimós S Barbi E. The decline of ‘deaths of despair’ in Italy: unveiling this phenomenon in a new context. Popul Health Metrics. (2026) 24:2. doi: 10.1186/s12963-025-00430-9, 41514439 PMC12801950

